# Extreme-Fungi and the Benefits of A Stressing Life

**DOI:** 10.3390/life9020031

**Published:** 2019-03-27

**Authors:** Laura Selbmann

**Affiliations:** 1 Department of Ecological and Biological Sciences, University of Tuscia, 01100 Viterbo, Italy; selbmann@unitus.it; Tel.: +39-0761-357012; 2 Italian National Antarctic Museum (MNA), Mycological Section, 16166 Genoa, Italy

A stress-free condition is considered for humans to be related to relaxation or happiness. Furthermore, all creatures constantly face threats in their life that must be met with adaptive responses, that invariably lead to enhanced vitality, vigor and resilience. Stress, therefore, might rather be regarded as a central concept for understanding life, adaptation, and evolution.

Recent advances in the world of extremophiles, namely microbial specialists of life under potent stressors, outstretched the concept of limits for life beyond any imagination. Nowadays, it has become evident that extreme environments are not a prerogative of archaea and bacteria: eukaryotes, and fungi in particular, are very skilled settlers in the extremes [1,2], doing even better than prokaryotes. In the Special Issue of Life “Fungi from Extreme Environments”, nine contributions reported diverse examples of fungi exploiting the extremes and explored some intriguing implications related to life at the edge, encompassing different fields of life science such as ecology, biodiversity, phylogeny, astrobiology, and biotechnological applications [3,4,5,6,7,8,9,10,11].

Fungi, the most versatile phylogenetic lineage for their stunning ecological and morphological plasticity, may easily explore and persist in novel environments, virtually dwelling in all types of habitats, even those normally precluded to most [12]. The first paper in the Issue by Connell et al. focused on this aspect, contributing to elucidate fungal diversity in Lake Fryxell [13], a permanent frozen water body located in one of the most inhospitable environments on Earth, the Antarctic McMurdo Dry Valleys. The authors explain how fungi may spread and disperse and indicate that they are the most diverse biota in Antarctica with a pivotal role in nutrient recycling and decomposition [14]. 

Another hotspot related to the extreme adaptation in fungi concerns the possible implication for the emergence of novel fungal pathogens [15,16]. This aspect was discussed by Marchetta et al., who reported on *Hortaea werneckii* as an example of halophilic fungus. The authors proceeded through a genetic study for intra-specific variability on a wide selection including strains from environmental samples (i.e., sea water, saltpans) and clinical strains. The study allowed them to clearly distinguish clinical and seawater groups, highlighting that the evolution of marine strains does not imply any clinical adaptation.

Extreme adaptation promotes adaptive radiation and speciation [17]. New species are frequently found when exploring new and unusual environments. This item was explored in the contributions by Ruibal et al. and Turchetti et al.; in the former, fungi in the highly extremotolerant group of Black Yeasts [18] were isolated from highly oxidant solar panels and new species are described here by multilocus phylogeny. Due their ability to form visible and biodeteriorating biofilms on exposed modern materials, the authors also discussed applicative implications for testing material. In the second paper, two basidiomycetous yeasts were described by multilocus phylogeny from cold environments, namely Arctic Svalbard and high altitude in the Alps. 

Furthermore, extremophiles are considered as model organisms in the search of extra-terrestrial life. Indeed, fungi from extreme environments may show a stunning resistance to stress, much higher, and sometimes not really related, to the injuries of their natural environment [19,20]. Onofri et al. reported a study evidencing molecular and structural integrity in the cells of two Antarctic fungi, *Cryomyces antarcticus* and *C. minteri* that were isolated in the Mars-like environment of the Antarctic McMurdo Dry Valleys, after a 1.5-year exposition in both space and Mars conditions. However, extremophilic fungi have implications in a number of applicative fields; their unusual degradative abilities, for instance, may be exploited for specific purposes as biocontrol or bioremediation [21]; the contribution by Spina et al. investigated the bioremediation potential of fungi adapted to pollutants isolated from landfill leachate and wastewaters. Extreme-fungi may also behave as a potent deteriorating agent even under unexpected conditions. Sterflinger et al. reported an extremely original study on the cultural heritage deteriorating potential of xerophilic fungi as the most frequent invaders of ancient pipe organs. Instead, the paper by Coleine et al. examined for the first time the pattern of functional groups in fungi living in Antarctic cryptoendolithic communities, considered a border lifestyle adopted by microbes before their extinction [22], collected under different environmental pressure due to altitude and sun exposure. The study confirmed lichenized taxa among the most abundant [23] and correlated the predominance of the functional group of Black Fungi, known for their exceptional extremotolerance, under higher environmental pressure. The contribution by Muggia and Grube was a comprehensive review of the recent advances in lichen symbioses studies and the advantages for spreading in the extremes for lichenized fungi.

Overall, among the endless aspects and implications related to the topic “Fungi from Extreme Environments”, the review and original articles by leading researchers in this dedicated Issue have significantly contributed to expand our knowledge of the intriguing world of extremophilic fungi, deeply discussing and reporting on remarkable examples of biodiversity, ecology, adaptation, survival, speciation, and applicative potential. 
